# Predicting hot spots in protein interfaces based on protrusion index, pseudo hydrophobicity and electron-ion interaction pseudopotential features

**DOI:** 10.18632/oncotarget.7695

**Published:** 2016-02-25

**Authors:** Junfeng Xia, Zhenyu Yue, Yunqiang Di, Xiaolei Zhu, Chun-Hou Zheng

**Affiliations:** ^1^ Key Laboratory of Intelligent Computing and Signal Processing of Ministry of Education, Institute of Health Sciences, Anhui University, Hefei, Anhui 230601, China; ^2^ Co-Innovation Center for Information Supply and Assurance Technology, School of Computer Science and Technology, Anhui University, Hefei, Anhui 230601, China; ^3^ School of Life Sciences, Anhui University, Hefei, Anhui 230601, China; ^4^ College of Electrical Engineering and Automation, Anhui University, Hefei, Anhui 230601, China

**Keywords:** hot spot, protrusion index, pseudo hydrophobicity, electron-ion interaction pseudopotential, cancer driver mutation

## Abstract

The identification of hot spots, a small subset of protein interfaces that accounts for the majority of binding free energy, is becoming more important for the research of drug design and cancer development. Based on our previous methods (APIS and KFC2), here we proposed a novel hot spot prediction method. For each hot spot residue, we firstly constructed a wide variety of 108 sequence, structural, and neighborhood features to characterize potential hot spot residues, including conventional ones and new one (pseudo hydrophobicity) exploited in this study. We then selected 3 top-ranking features that contribute the most in the classification by a two-step feature selection process consisting of minimal-redundancy-maximal-relevance algorithm and an exhaustive search method. We used support vector machines to build our final prediction model. When testing our model on an independent test set, our method showed the highest F1-score of 0.70 and MCC of 0.46 comparing with the existing state-of-the-art hot spot prediction methods. Our results indicate that these features are more effective than the conventional features considered previously, and that the combination of our and traditional features may support the creation of a discriminative feature set for efficient prediction of hot spots in protein interfaces.

## INTRODUCTION

Protein-protein interactions (PPIs) play a critical role in nearly all aspects of cellular functions like DNA replication and signal transduction [1, 2]. Studies of principles governing PPIs have revealed that most of the binding free energy in an interaction is contributed by a small fraction of interface residues known as hot spots [3]. Identifying these hot spot residues in protein interfaces can help us better understand protein binding mechanisms and may also help us to modulate protein-protein association. In addition, it has been suggested that mutations in protein interfaces play a key roles in cancer development [4], which means that systematically studying the roles of mutations in protein interfaces, including hot spots, can help us to identify cancer driver genes. For example, Porta-Pardo *et al.* [4] have explored the role of missense mutations on PPI interfaces as cancer driver mutations in a pan-cancer cohort of 5,989 tumors from 23 projects of The Cancer Genome Atlas. Their analysis identified PPI interfaces enriched in somatic mutations in a total of 103 genes, proving that alteration of interaction interfaces is a common pathogenic mechanism of cancer mutations.

Experimentally, hot spots can be detected using molecular biology and thermodynamic methods upon site-directed mutagenesis like alanine scanning, which aims to evaluate the change in the binding free energy resulting from the mutations of protein residues to alanine within a protein interface. A database collecting a list of hot spots identified by alanine scanning mutagenesis experiments is Alanine Scanning Energetics Database (ASEdb) [5]. Binding Interface Database (BID) is another database which contains experimentally verified hot spots from literature studies [6].

The characteristics of hot spots have been extensively studied. Sequential and structural analysis of the protein-protein interface has indicated that hot spots are more conserved than non-hot spot residues [7, 8]. Also, it reveals that hot spots are usually tightly packed within the interface and surrounded by a ring of energetically less important residues that occlude bulk solvent from the hot spots [9]. Other features are also found to be correlated with hot spots, including pairing potential [10] and protrusion index [11].

Because identification of hot spots by experimental methods like alanine scanning mutagenesis is time-consuming, labor-intensive and expensive, there is a need for developing reliable computational methods to identify hot spots. In recent years, a number of computational methods have been proposed which exploit one or more properties of hot spots, as described above, to detect them from protein interfaces. Some of these methods are based on molecular dynamics simulation techniques [12–14] while others are based on different energy functions such as Robetta [15] and FOLDEF [16]. Recently, there has been considerable progress in applying machine learning approaches to discriminate hot spots from the rest of the interface residues such as decision trees [17], neural network [18], support vector machine [19], random forests, and extreme learning machine. We also have developed two hot spot prediction approaches based on support vector machine, i.e. APIS [11] and KFC2 [20], which have both clearly improved predictive ability. These machine learning methods try to predict hot spots by using sequence, structure or a combination of both sequence and structure information. Most of these approaches are trained on a subset of ASEdb and tested independently on another dataset obtained from BID. Overall, these computational approaches, especially machine learning methods, have become a valuable complement to experimental approaches and can reduce the number of mutations that experimental researchers have to pursue when attempting to establish principles about binding mechanisms.

**Figure 1:**
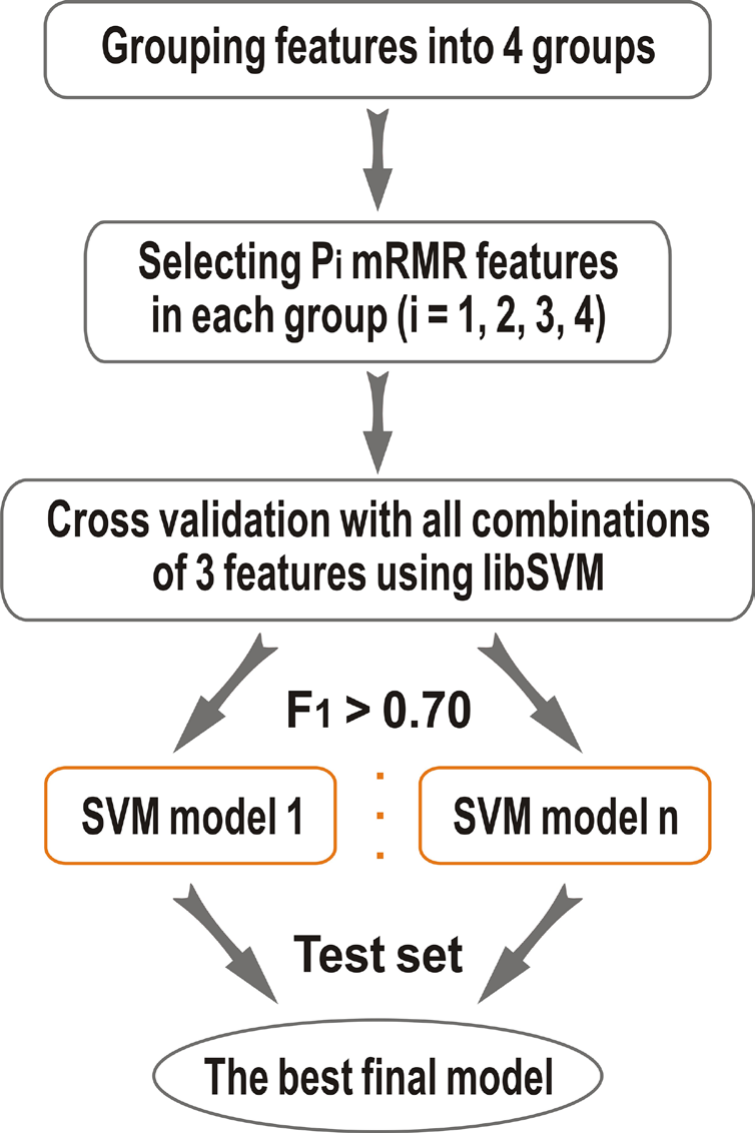
The framework of the present HEP method

In this paper, we proposed a novel hot spot prediction method, HEP, which based on our previous APIS and KFC2 approaches. First, we constructed a wide variety of 108 sequence, structural, and neighborhood features to characterize potential hot spot residues, including conventional ones and new ones exploited in this study. Then, we used a two-step feature selection method to identify the best top-ranking features that contribute the most in the classification. Finally, we built a model (HEP) using support vector machine (SVM) and evaluated the performance of the proposed HEP model on two benchmark datasets, ASEdb and BID. The results show that the proposed method was able to outperform existing hot spot prediction methods. The basic architecture of the proposed model HEP is shown in (Figure 1).

## RESULTS

### Feature selection and predictive ability

Features for machine learning methods is an important factor in building a model. In this work, we investigated physicochemical, structural, and neighborhood features for identifying hot spot (shown in Table 1 and Supplementary Table S1). Additional features [11] derived from residue-residue pairing preferences at the interface, residue conversation and temperature factor are also considered in HEP. The physicochemical features consist of a total of 17 attributes, where PSHP (pseudo hydrophobicity) is a novel feature for hot spot identification. The structural features include accessible surface area (ASA), relative ASA (RASA), depth index (DI), protrusion index (PI) [11], and the residue's position information within the protein interface [20]. Because the environment of a target interface residue can affect its importance to the protein interaction, we defined 33 features related to neighbors of the target residue, which were used in our KFC2 hot spot model [20] and DBSI DNA-binding site model [21]. In total, we identified 4 groups of features, which consist set of 108 feature vectors for each hot spot. More details about the feature encoding schema can be found in features representation under Materials and Methods section.

**Table 1:** The composition of each feature group

Group name	The number of features*	Feature type
Group 1	17 (6)	Physicochemical feature
Group 2	55 (18)	Structural feature
Group 3	33 (11)	Neighborhood feature
Group 4	3 (1)	Other feature

* The number within the parenthesis represents the number of features after the two-step feature selection's first step (mRMR method).

To assess the feature importance of the 108 features in predicting hot spots, we applied a two-step feature selection method on ASEdb Dataset. In the first step, we used minimum Redundancy Maximum Relevance (mRMR) [22, 23], a well-designed filter method, to assess the feature vector elements in each feature group (physicochemical features, structural features, neighborhood features, and features derived from residue-residue pairing preferences at the interface, residue conversation and temperature factor). The top 1/3 features from different groups were selected. The total number of features we got is 36. Table 1 gives the information of the composition of each feature group and the number of features that were selected.

In the second step, we used a similar wrapper method in our previous work. We first considered 2 different features combinations. Then we added one feature to train our models with SVM each time. This was an effective way to decrease the combinational complexity of feature sampling while creating models with diverse feature combinations. According to the models trained using different feature combinations, we found that the following three features achieved the best model among those we examined: electron-ion interaction pseudopotential (EIIP), pseudo hydrophobicity (PSHP), and relative change in total mean PI upon binding (RctmPI). Many more features and combinations were tried, but no better models were obtained. For details of feature representation and selection, please see features representation and two-step features selection under Materials and Methods section.

After identifying the best feature combination, we wanted to optimize the corresponding parameters for SVM. The LIBSVM package [24] was employed in this work. The Gaussian Radial Basis Function kernel was exclusively used in the computations based on previous studies. The capacity parameter C (0.0–80.0) and the kernel parameter G (0.0–2.0) of the SVMs were tried using a grid search method for Gaussian RBF kernel functions. The cross-validation produced the best results using parameter values C = 80.0 and G = 0.002. These parameters were then used to train the final HEP model on the entire training set (ASEdb), on which a 10-fold cross-validation was performed to assess predictive performance.

The performance of our model is measured by six metrics: accuracy (ACC), specificity (SPE), precision (PRE), recall (REC), MCC and F1 score. F1-score is the harmonic mean of the precision and recall, which is widely used to handle unbalanced data such as hot spot data. Table 2 illustrates the results of cross-validations. HEP achieved Accuracy = 0.73, Specificity = 0.70, Precisio *n* = 0.63, Recall = 0.77, F1 = 0.70, and MCC = 0.46. We can see that compared with HEP model, F1 score is quite low when we use all 108 features without feature selection. After feature selection, we got 3 significant features. We predict 48 of the hot spots correctly with 28 false positives. On the other hand, 64 of the non-hot spots are correctly classified with 14 false negatives.

**Table 2:** Prediction results of the cross-validation with different feature number

Feature used	TP	TN	FP	FN	ACC	SPE	PRE	REC	F1	MCC
All	5	90	2	57	0.62	**0.98**	**0.71**	0.08	0.14	0.14
HEP	48	64	28	14	**0.73**	0.70	0.63	**0.77**	**0.70**	**0.46**
-EIIP	35	77	15	27	**0.73**	0.84	0.70	0.56	0.63	0.42
-RctmPI	25	74	18	37	0.64	0.80	0.58	0.40	0.48	0.23
-PSHP	46	67	25	16	**0.73**	0.73	0.65	0.74	0.69	**0.46**

The highest values are highlighted in bold.

We have further tested the effect of deleting one feature from the 3 features (EIIP, PSHP, and RctmPI). We observe that the specificity and precision increases but the recall decreases when the feature EIIP was removed. In other words, fewer positive hot spot residue are predicted with higher percentage of true cases. Compared with HEP model, a slight decrease in F1-score performance from 0.70 to 0.69 is observed when the feature PSHP was removed. Further, when the feature RctmPI was eliminated, both the precision and recall decrease, with the F1-score of only 0.48. The results indicate that the performance based on 2 features (maximum F1 = 0.69) was lower than HEP model based on the three features EIIP, PSHP, and RctmPI. Therefore, we used these three features based classifiers as our final model to infer hot spot residues in protein interfaces.

### Performance comparisons with other prediction methods

We have been careful in making comparisons between models, and we have provided exhaustive information to facilitate future comparisons with the HEP model on the independent test dataset. The detailed measures of these different predictors are listed in Table 3. We can see that our method substantially outperforms the existing methods in four performance metrics (accuracy, recall, F1 score and MCC). We have the highest REC, which means our method can predict more hot spots than the others. Furthermore, the F1-score of our method is 0.70, while those of the existing methods fall in the range of 0.34–0.64. Comparing with APIS and KFC2, we find our performance has a high improvement. The predictive results for the independent test also indicate that the proposed model performs significantly better than the existing state-of-the-art approaches.

**Table 3:** Comparison of different hot spot prediction methods in the independent test set

Methods	TP	TN	FP	FN	ACC	SPE	PRE	REC	F1	MCC
HEP	32	68	21	6	**0.79**	0.76	0.60	**0.84**	**0.70**	**0.56**
APIS	28	67	21	11	0.75	0.76	0.57	0.72	0.64	0.45
Robetta	12	80	11	24	0.72	0.88	0.52	0.33	0.41	0.25
FOLDEF	10	78	11	28	0.69	0.88	0.48	0.26	0.34	0.17
KFC	12	75	13	27	0.69	0.85	0.48	0.31	0.38	0.19
MINERVA	17	79	9	22	0.76	**0.90**	**0.65**	0.44	0.52	0.38
KFC2a	29	64	24	10	0.73	0.73	0.55	0.74	0.63	0.44
KFC2b	21	77	12	17	0.77	0.87	0.64	0.55	0.60	0.44

The highest values are highlighted in bold.

### Case studies

#### Complex between the erythropoietin receptor and erythropoietin mimetic peptide

**Figure 2:**
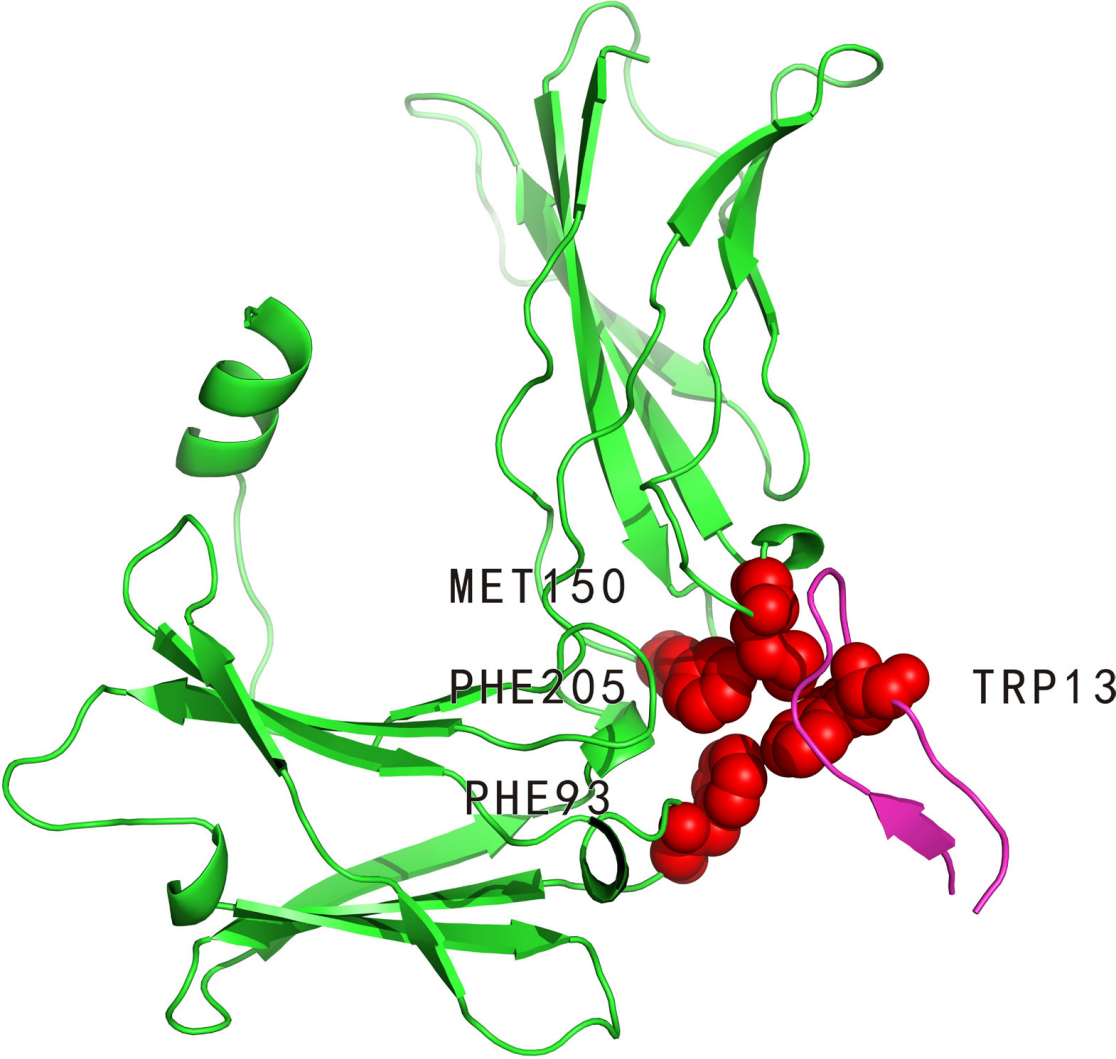
Interaction between erythropoietin receptor (PDBID: 1ebp, chain A, coloured by green) and erythropoietin mimetic peptide (PDBID: 1ebp, chain C, coloured by magenta) PHE93: A, PHE205: A, MET150: A and TRP13: C (represented by VDW spheres) are experimentally determined hot spots in the 1ebpAC interface. Of these four residues, PHE93: A, MET150: A and TRP13: C (all coloured by red) were correctly predicted by our method.

The erythropoietin receptor (PDBID: 1ebp, chain A) binds to erythropoietin mimetic peptide (PDBID: 1ebp, chain C). Four hot spots (PHE93: A, PHE205: A, MET150: A and TRP13: C, indicated in (Figure 2)) and five non-hot spots have been experimentally determined in the 1ebpAC interface. In these 9 alanine-mutated residues, our method identified three residues (PHE93: A, MET150: A and TRP13: C) as hot spots and the rest as non-hot spots. Three of the four hot spots were correctly predicted. In contrast, APIS predicted only one residue (TRP13: C) as hot spot and the others as non-hot spots.

#### Complex between the beta-catenin and adenomatous polyposis protein

**Figure 3:**
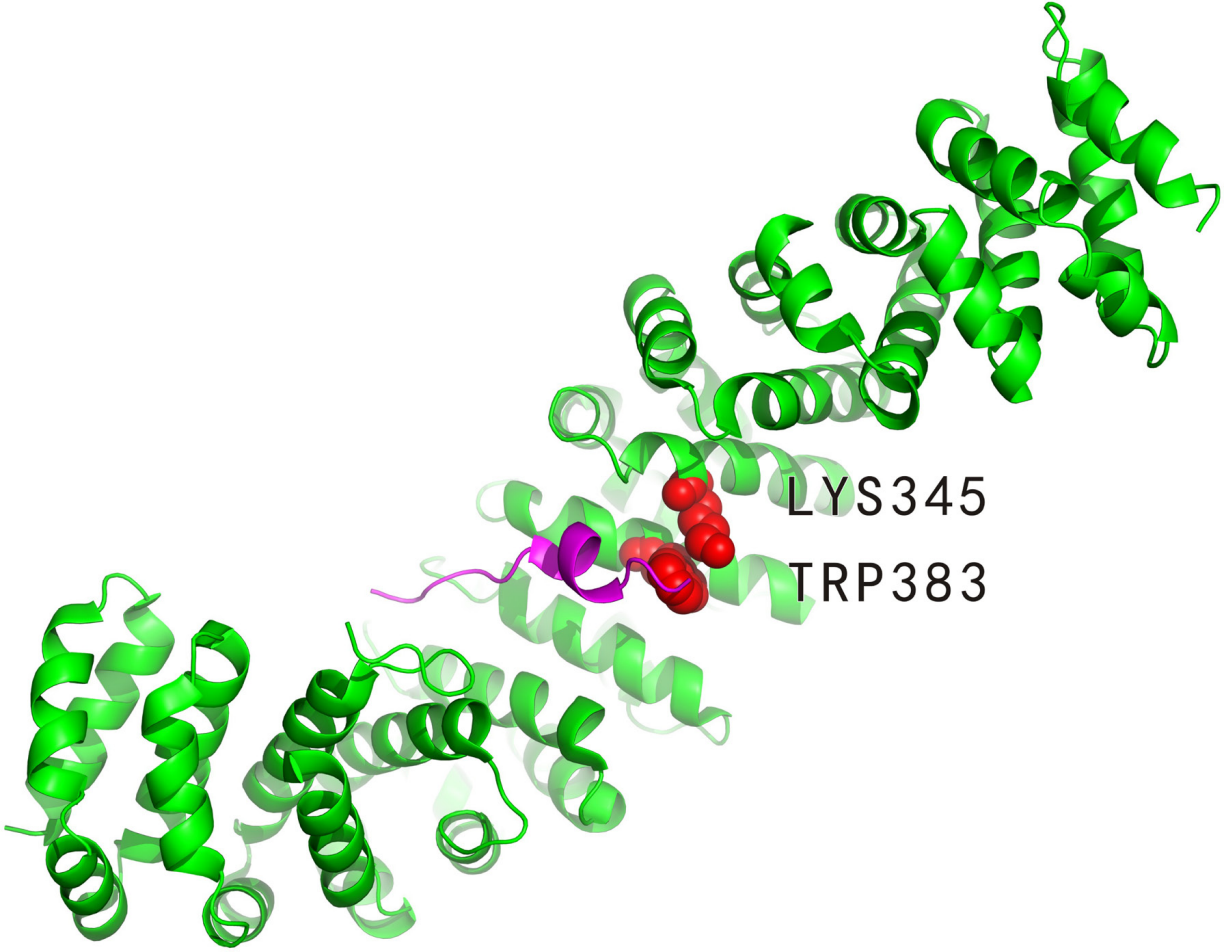
Interaction between the beta-catenin (PDBID: 1jpp, chain B, coloured by green) and adenomatous polyposis protein (PDBID: 1jpp, chain D, coloured by magenta) The defined hot spot residues are LYS345: B and TRP383: B (represented by VDW spheres) in the 1jppBD interface. TRP383: B (coloured by red) is the hot spot that was correctly predicted by our method.

The beta-catenin (PDBID: 1jpp, chain B) binds to adenomatous polyposis protein (PDBID: 1jpp, chain D). Experimentally identified hot spots residues at 1jppBD interface are LYS345: B and TRP383: B (indicated in Figure 3). Furthermore, LYS354: B, ARG386: B, LYS435: B, ARG469: B and HIS470: B were experimentally determined to be non-hot spots. Our method correctly predicted one out of the two hot spot residues, that is, TRP383: B, and all the non-hot spots. As a comparison, APIS correctly predicted all the non-hot spots but none hot spots.

## DISCUSSION

**Figure 4:**
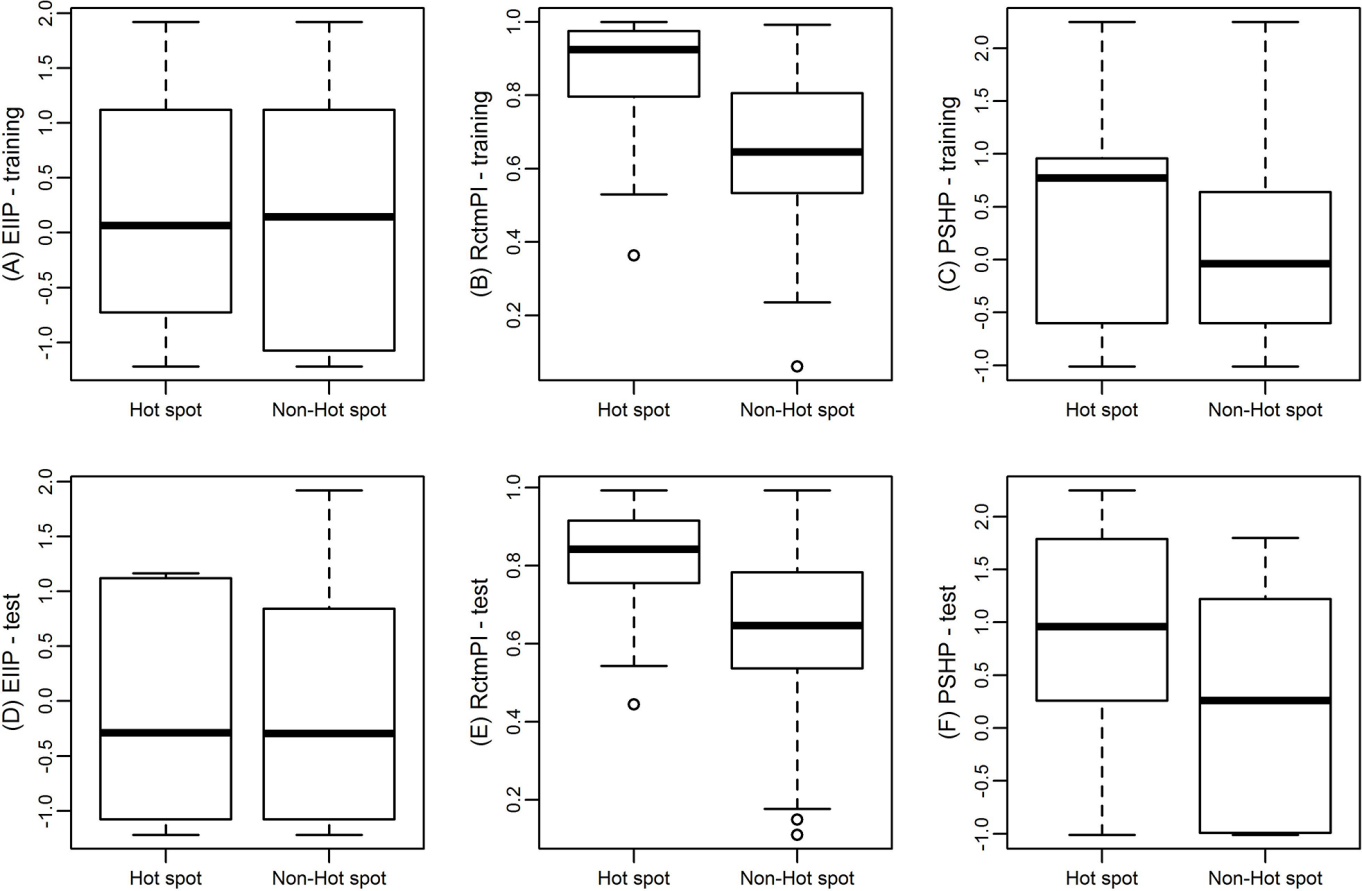
Box plot of hot spots and non-hot spots with respect to their EIIP (A), RctmPI (B), and PSHP (C) in training data, and EIIP (D), RctmPI (E), and PSHP (F) in test data, respectively In each box, the bottom and the top of the box are the lower and upper quartiles, respectively, and the middle line is the median.

In this study, we computed 108 different features including physicochemical features, structural features and other conventional features. We used a two-step feature selection method to choose significant features. The importance of features provides insights for their discrimination abilities between hot spots and non-hot spots. In order to show different ability of the three features for distinguishing hot spots from non-hot spots, statistical analysis method Mann-Whitney *U* test (the confidence level is 0.95) is performed. The differences of the three features between hot spots and non-hot spots are significant shown in (Figure 4). And Table 4 gives the median value and *P*-value of hot spots and non-hot spots for these 3 features.

**Table 4:** Statistic data of hot spots and non-hot spots in training and test data set

Feature	Data	Median value (hot spots)	Median value (non-hot spots)	*P*-value
EIIP	Training	0.065	0.144	0.714
	Test	−0.291	−0.296	0.598
RctmPI	Training	0.924	0.644	0.000
	Test	0.842	0.647	0.000
HP	Training	−0.610	−0.040	0.728
	Test	0.960	−0.040	0.014
PSHP	Training	0.770	−0.040	0.009
	Test	0.960	0.260	0.003
CHP	Training	0.960	0.260	0.000
	Test	1.010	0.990	0.010

(Figure 4A) shows the box plot of EIIP between hot spots and non-hot spots in the training set. The median value of EIIP of hot spots and non-hot spots is 0.0646 and 0.1442, respectively with a *P*-value of 0.714 (Table 4). According to the *P*-value, EIIP may not be a good feature to distinguish hot spots from non-hot spots by itself. However, in Nguyen *et al.* [25]. They use frequency-related features involved physicochemical characteristic such as electron-ion interaction potentials and ionization constants to predict hot spots and get a high F1. Their results brought evidence to support the conjecture of Cosic *et al.* [26]. That protein hot spots are associated with frequency features of physicochemical characteristics of the amino acid sequence. Meanwhile, we tested the result without the feature using the same method and got the best F1 of 0.52 and the best recall of 0.61, which were far from our performance. It means that EIIP is an important feature even that it has not good ability to discriminate hot spots and non-hot spots when used alone.

(Figure 4B) is the box plot of RctmPI, in which the median value of RctmPI of hot spots (0.9237) is higher than that of non-hot spots (0.6442). In addition, the *P*-value is 0 (Table 4). These results suggest the hot spots residues prefer to protrude from the surface, which are in accordance with the results obtained from Pintar *et al.* [27]. and Wu *et al.* [28]. So the feature RctmPI is a useful feature to construct the SVM model. In our previous work [11], we used individual feature to construct models and also found the individual-feature model with RctmPI has more prediction power.

(Figure 4C) represents the box plot of PSHP of hot spots and non-hot spots. The median PSHP of hot spots is 0.77 and that of non-hot spots is −0.04 (*P*-value = 0.009, Table 4). Thus, PSHP is also an important feature for distinguishing hot spots from non-hot spots. To consider the relationship among PSHP, residue hydrophobicity (HP), and residue hydrophobicity multiplying charges (CHP), we tested our results by using them respectively with the other two features (EIIP and RctmPI). We also plotted their box plots as that showed in (Supplementary Figure S1). CHP is defined as the hydrophobicity multiplied by −1 when the residue is ASP, GLU, LYS or ARG and the hydrophobicity when the residue is other type, which is a little different from that defined in PSHP. (Supplementary Figure S1B) shows the box plot of HP of hot spots and non-hot spots. The median HP of hot spots is −0.61 and that of non-hot spots is −0.04, with a *P*-value of 0.728, respectively. (Supplementary Figure S1C) is the box plot of CHP with a median value 0.96 for hot spots and 0.26 for non-hot spots (*P*-value = 0). The charged residues, ASP, GLU, LYS and ARG, can often be hot spots when they are in the interface, because they formed strong hydrogen bond or salt bridges with other residues. We multiplied the hydrophobicity scales of these residues by −1 to make the correspondence between these residues with other hydrophobic residues (TRP, PHE) that are often hot spots in the interface. However, it turned out LYS and ARG in the interface could have been well described by other two features. We owe it to that the side chains of ASP and GLU are long but not too long compared to LYS and ARG, so that when they are in the interface the entropy loss can be complementary by the strong hydrogen bond or salt bridges formed by them with other residues. (Figure 4D–4F) also shows the box plots of hot spots and non-hot spots in the test data set.

In conclusion, hot spot residues comprise a small fraction of the interface residues that make a dominant contribution to the free energy of the binding. Owing to the time consumption and labor intensity in experiment determination of binding free energy for alanine-mutated residues, computational methods can thus be helpful in suggesting residues for possible experimentation. In this study, we proposed a new computational method to identify hot spots in the protein interfaces. We extracted 108 various features from different aspects such as physicochemical, structure, atom contact from APIS and KFC2. Then we used a two-step feature selection approach that can significantly improve the prediction performance and reduce the risk of over-fitting. Firstly, we used mRMR to select a number of features from different feature groups, which can facilitate the integration of other feature selection methods to select a compact subset of superior features within a very low cost. Secondly, we tried to use the least number of features to construct our model and we selected only three features at last when we tried different feature combinations. Test results on independent data set indicated that our method is more effective than the major existing hot spot prediction methods. Researchers who are interested in finding new features of hot spot residues could use the HEP model to characterize the roles of their features. HEP would also benefit from these new features on the other hand.

## MATERIALS AND METHODS

### Datasets

Two benchmark hot spot datasets are used in this work. Following our previous work [11], we used the ASEdb dataset [5] and the published data of Kortemme and Baker [15] as the training set. An interface residue in the dataset corresponding to a binding free energy higher or equal to 2.0 kcal/mol is defined as a hot spot residue. The interface residues with binding free energy less than 0.4 kcal/mol is considered as non-hot spot. Other interface residues with binding free energy between 0.4 and 2.0 are excluded from the training set for the purpose of increasing discrimination between hot spots and non-hot spots. The final training set comprised of 62 hot spots and 92 non-hot spots.

Similar to our previous work [11], the BID database [6] was used an as independent test set. In BID, the alanine mutation data are categorized as “strong”, ”intermediate”, “weak”, or “insignificant”. In our study, only the residues labeled as “strong” are considered as hot spots and the remaining residues are regarded as non-hot spots. As a result, the final test set contained 127 alanine mutated interface residues in 18 complexes with 39 hot spots and 88 non-hot spots. For details of these two datasets, please refer to [11].

### Features representation

A wide variety of 108 physicochemical, structural and neighbor features are used to characterize potential hot spot residues. In our study, we combined the features used in APIS [11] and KFC2 [20]. Meanwhile, we added a number of features proposed in DBSI [21] in this study. These features can be roughly divided into four groups: (I) Physicochemical features; (II) structural Features related to solvent accessibility; (III) features related to neighbors of the target residue; and (IV) other features.

### Physicochemical features

Physicochemical features of an amino acid residue were described by 16 values such as hydrophobicity, hydrophilicity, isoelectric point, mass, expected number of contacts with 14 Å sphere, electron-ion interaction pseudopotential (EIIP). The description of these features can be found in our previous works [11, 20].

In addition, a new feature called pseudo hydrophobicity (PSHP) was generated based on the combination of hydrophobicity and charge. If the charge of the residue was non-negative, the pseudo hydrophobicity was defined as the hydrophobicity of the residue; if the charge of the residue was negative, i.e. Asp or Glu, the pseudo hydrophobicity was defined as the product of the hydrophobicity index and the charge of the residue. Here the negatively charged residues were assigned a charge of −1.

### Features extracted from structural attributes

55 structure-based features related to accessible surface area (ASA), relative ASA (RASA), depth index (DI), and protrusion index (PI) were used in this study. These features were calculated using PSAIA [29]. For details, please refer to our previous works [11, 20].

### Features related to neighbors of the target residue

The environment of a target interface residue is very important because hot spots were found to be clustered within tightly packed regions. We defined environmental features of a target residue with two distances cutoffs 4.0 and 5.0 Å. The number of residues and the number of atoms around the side chain of the target residue were calculated within this environment, respectively. We also calculated the number of rotatable single bonds within the side chain. In addition, the weighted rotatable single bond number, which is the number of rotatable single bonds divided by the number of atoms in the side chain, was also calculated. Lastly, we calculated the total hydrophobicity of residues around the side chain of the target residue, with the purpose of reflecting the environmental hydrophobicity of the target residues.

### Other features

B-factor was used to represent the flexibility of amino acid residue. Secondary structure of each residue collected from DSSP [30] was also used to describe hot spots. We also computed the pair potential of each residue in our data set by using the method in Xia at el [11]. Additionally, the evolution rate for each residue was obtained using the Rate4Site algorithm [31], which was implemented in the ConSurf-DB [32] server.

Totally, 108 descriptors were generated for each interface residue (For details, please see Supplementary Table S1).

### Two-step features selection

Feature selection, more precisely feature subset selection, is performed to eliminate uninformative properties to generate robust and general prediction models. In this work, we propose a two-step feature selection method, as shown in (Figure 1), to select a subset of features, with which to obtain better discrimination of hot spots and non-hot spots.

In the first step, we divided the feature into 4 groups. Then we assessed the feature elements using minimum Redundancy Maximum Relevance (mRMR) [22, 23] in each group. mRMR is very useful in the preprocess of feature selection as described in Peng *et al.* [22]. We applied mRMR to each group and chose the top 1/3 features of group 1, 2, 3, and 4. Table 1 gives the information of the composition of each group and the number of features that were selected and non-selected. After that, we got 36 features.

In our second step, we used the similar method in our previous work [20]. We first selected 2 features from the selected 36 features. Then we added one feature each time until the F1 score and MCC was improved. And we used support vector machines (SVM) to evaluate the features by 10-fold cross-validation. After that, we combined features from different classes to create diverse feature combination sets.

### Classification method

The classification model for predicting hot spots was based on SVM, which is a class of effective supervised learning methods that demonstrate high prediction accuracy while efficiently avoiding the overfitting problem. The LIBSVM package (Chang and Lin 2001) was employed in this work. The Gaussian Radial Basis Function kernel was exclusively used in the computations based on previous studies. We tried different C factors (ranging from 0 to 80) and different G factors (ranging from 0 to 2) for Gaussian RBF kernel functions. The value of C controls the trade-off between allowing training errors and forcing strict margins, while the value of G determines the Gaussian RBF width. Depending on the composition of the data set and the number of features used, the optimal values for C and G can vary tremendously.

### Performance assessment

Predicting a binding site as hot spot or non-hot spot is a binary classification problem, and many measures have been introduced for validation issues. Here the prediction performances are evaluated by the overall prediction accuracy (ACC), specificity (SPE), precision (PRE), recall (REC), F1 score, and the Matthew's correlation coefficient (MCC). These measures are defined as,

$$\begin{array}{lll} \text{ACC} & = & \left(\text{TP}+\text{TN})/(\text{TP}+\text{FP}+\text{TN}+\text{FN}\right)\\ \text{SPE} & = & \text{TN}/(\text{TN}+\text{FP})\\ \text{PRE} & = & \text{TP}/(\text{TP}+\text{FP})\\ \text{REC} & = & \text{TP}/(\text{TP}+\text{FN})\\ \text{F}1 & = & 2\times \text{REC}\times \text{PRE}/(\text{REC}+\text{PRE})\\ \text{MCC} & = & \frac{\left(TP\times TN-FP\times FN\right)}{\sqrt{\begin{array}{l} \left(\text{TP}+\text{FN})(\text{TP}+\text{FP}\right)\\ \left(\text{TN}+\text{FP})(\text{TN}+\text{FN}\right) \end{array}}} \end{array}$$

where TP, FP, TN, and FN are the number of true positive (correctly predicted hot spot), false positive (non-hot spot residue incorrectly predicted as hot spot), true negatives (correctly predicted non-hot spot), and the number of false negatives (hot spot residue incorrectly predicted as non-hot spot), respectively.

## SUPPLEMENTARY MATERIALS FIGURE AND TABLE


