# Supplementary material for: Predicting hot spots in protein interfaces based on protrusion index, pseudo hydrophobicity and electron-ion interaction pseudopotential features
Source: Oncotarget. 2016 Feb 25;7(14):18065–75. doi: 10.18632/oncotarget.7695 (PMC4951271; doi:10.18632/oncotarget.7695)
Supplement: Supplementary file 1 [file oncotarget-07-18065-s001.pdf]

Predicting hot spots in protein interfaces based on protrusion index, pseudo hydrophobicity and electron-ion interaction pseudopotential features

Supplementary Materials

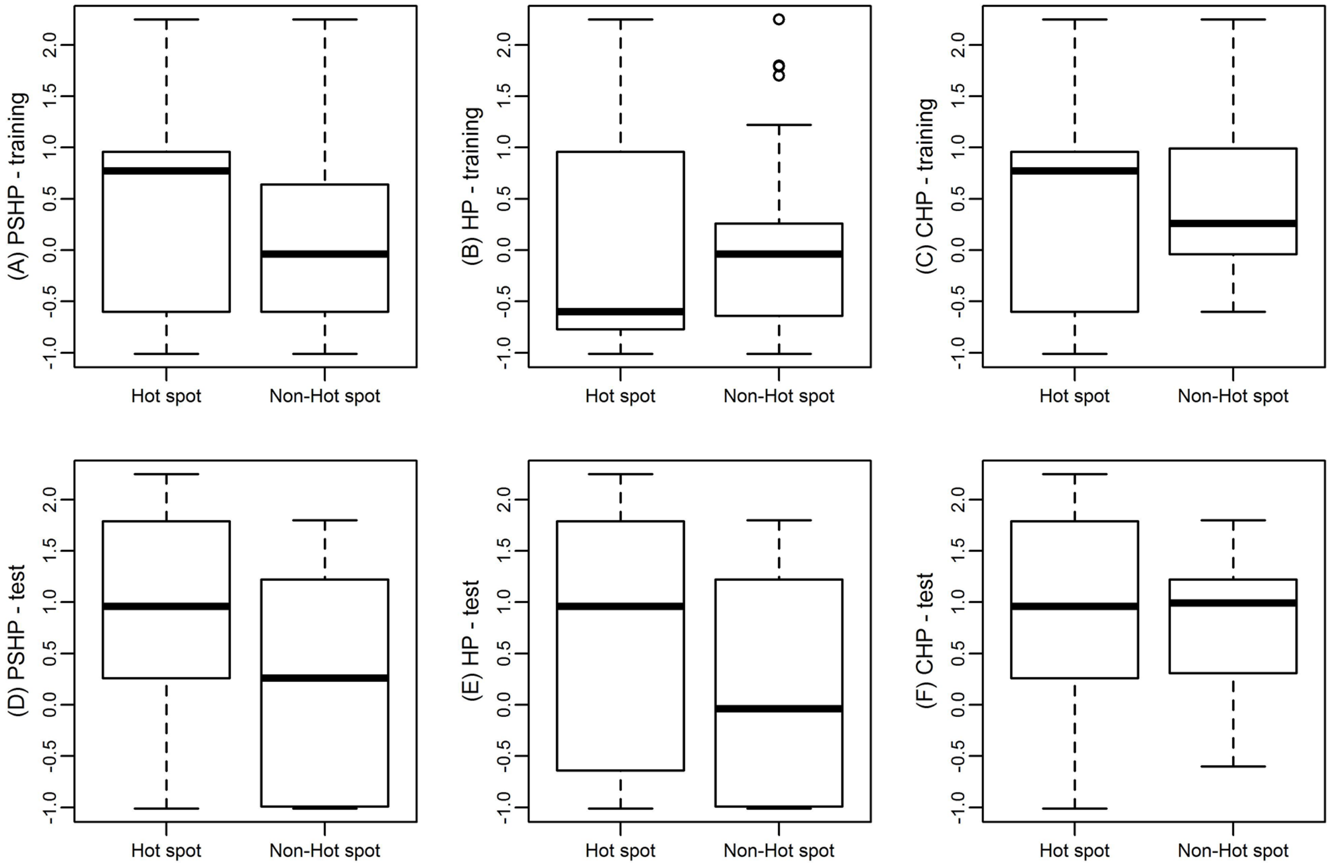

Supplementary Figure S1: Box plot of hot spots and non-hot spots with respect to their PSHP (A), HP (B), and CHP (C) in training data, and PSHP (D), HP (E), and CHP (F) in test data, respectively. In each box, the bottom and the top of the box are the lower and upper quartiles, respectively, and the middle line is the median.

Supplementary Table S1: A complete list of features used in training the HEP model

Feature type	Name	Description
Physicochemical features (17)	HP	Hydrophobicity
	PSHP	Pseudo hydrophobicity
	HPL	Hydrophilicity
	MASS	Mass
	CHARGE	Charge
	PS	Propensity
	SIZE	Maximum solvent accessible surface area
	IP	Isoelectric point
	NA	Numbers of atoms
	NEC	Number of electrostatic charge
	ENC	Expected number of contacts within 14 Å sphere
	EIIP	Electron-ion interaction pseudopotential
	NPHP	Number of potential hydrogen bonds
	DELTA_HP	The change of hydrophobicity in original residue and mutated residue
	DELTA_CHARGE	The change of charge in original residue and mutated residue
	DELTA_SIZE	Relative change of residue size in original residue and mutated residue upon mutated residue
	PRODUCT	The combination of DELTA_HP, DELTA_CHARGE, and DELTA_SIZE
Structure-based features (55)	UtASA	Unbound total ASA (accessible surface area)
	UbASA	Unbound backbone ASA
	UsASA	Unbound side-chain ASA
	UpASA	Unbound polar ASA
	UnASA	Unbound non-polar ASA
	UtRASA	Unbound total RASA (relative ASA)
	UbRASA	Unbound backbone RASA
	UsRASA	Unbound side-chain RASA
	UpRASA	Unbound polar RASA
	UnRASA	Unbound non-polar RASA
	UtmDI	Unbound total mean DI (depth index)
	UsmDI	Unbound side-chain mean DI
	UmaxDI	Unbound maximum DI
	UminDI	Unbound minimal DI
	UtmPI	Unbound total mean PI (protrusion index)
	UsmPI	Unbound side-chain mean PI
	UmaxPI	Unbound maximum PI
	UminPI	Unbound minimal PI
	BtASA	Bound total ASA

Structure-based features (55)	BbASA	Bound backbone ASA
	BsASA	Bound side-chain ASA
	BpASA	Bound polar ASA
	BnASA	Bound non-polar ASA
	BtRASA	Bound total RASA
	BbRASA	Bound backbone RASA
	BsRASA	Bound side-chain RASA
	BpRASA	Bound polar RASA
	BnRASA	Bound non-polar RASA
	BtmDI	Bound total mean DI
	BsmDI	Bound side-chain mean DI
	BmaxDI	Bound maximum DI
	BminDI	Bound minimal DI
	BtmPI	Bound total mean PI
	BsmPI	Bound side-chain mean PI
	BmaxPI	Bound maximum PI
	BminPI	Bound minimal PI
	RctASA	Relative change in total ASA upon complexation
	RcbASA	Relative change in backbone ASA upon complexation
	RcsASA	Relative change in side-chain ASA upon complexation
	RcpASA	Relative change in polar ASA upon complexation
	RcnASA	Relative change in non-polar ASA upon complexation
	RctmDI	Relative change in total mean DI upon complexation
	RcsmDI	Relative change in side-chain mean DI upon complexation
	RcmaxDI	Relative change in maximum DI upon complexation
	RcminDI	Relative change in minimal DI upon complexation
	RctmPI	Relative change in total mean PI upon complexation
	RcsmPI	Relative change in side-chain mean PI upon complexation
	RcmaxPI	Relative change in maximum PI upon complexation
	RcminPI	Relative change in minimal PI upon complexation
	delASA	The change in ASA between monomer and complex (delta ASA)
	RES_WT	The ratio of residue's delASA and delASA in all interface residues
	PER	A residue's position at the protein-protein interface
	POS_PER	A second measure of position within the protein interface
	nPOS_PER	100 - POS_PER
	nPER	The ratio of complex's ASA and monomer's ASA

Features related to neighbors of the target residue (33)	CH_T	Total charge of neighbor residues (distance cut off = 7)
	HP_T	Total hydrophobicity of neighbor residues (distance cut off = 7)
	ROT_T	Total rotatable single bond of neighbor residues (distance cut off = 7)
	WT_ROT_T	Total weighted rotatable single bond of neighbor residues (distance cut off = 7)
	AVE_CH	CH_T/RES_T (distance cut off = 7)
	AVE_HP	HP_T/RES_T (distance cut off = 7)
	AVE_ROT	ROT_T/RES_T (distance cut off = 7)
	AVE_WT_ROT	WT_ROT_T/RES_T (distance cut off = 7)
	AVE_SVAL	SS_VAL/RES_T (distance cut off = 7)
	ROT	Total rotatable single bond of neighbor residues (distance cut off = 4)
	WT_SC	The score of the weight (distance cut off = 4)
	nROT	ROT*WT_SC
	RESN	Residue's number (distance cut off = 4)
	ATMN	Atom's number (distance cut off = 4)
	HPD	Hydrophobicity (distance cut off = 4)
	SC1	Score1 of the around residues (cutoff = 4,5.0,5.5)
	SC2	Score2 of the around residues (cutoff = 4,5.0,5.5)
	SC3	Score3 of the around residues (cutoff = 4,5.0,5.5)
	SC4	Score4 of the around residues (cutoff = 4,5.0,5.5)
	SC5	Score5 of the around residues (cutoff = 4,5.0,5.5)
	SC6	Score6 of the around residues (cutoff = 4,5.0,5.5)
	SC7	Score7 of the around residues (cutoff = 4,5.0,5.5)
	SC8	Score8 of the around residues (cutoff = 4,5.0,5.5)
	SC9	Score9 of the around residues (cutoff = 4,5.0,5.5)
	SC10	Score10 of the around residues (cutoff = 4,5.0,5.5)
	SC11	Score11 of the around residues (cutoff = 4,5.0,5.5)
	SC12	Score12 of the around residues (cutoff = 4,5.0,5.5)
	SC13	Score13 of the around residues (cutoff = 4,5.0,5.5)
	SC14	Score14 of the around residues (cutoff = 4,5.0,5.5)
	NSCORE	100.0*float(nrot)/float(atmn)/float(maxASAsc)
	NSCORE1	100.0*float(nrot1)/float(atmn1)/float(maxASAsc)
	NSCORE2	100.0*float(nrot2)/float(atmn2)/float(maxASAsc)
	AVE_SSIM	SS_SIM/RES_T (distance cut off = 7)
Other features (3)	PP	Pair potential
	TF	Temperature factor (B-factor)
	RC	Residue conservation
